# Structure of a push–pull olefin prepared by ynamine hydro­boration with a borandiol ester

**DOI:** 10.1107/S2056989020005289

**Published:** 2020-04-21

**Authors:** Joël Gubler, Peter Chen

**Affiliations:** a Laboratorium für Organische Chemie, ETH Zürich, Zürich, Switzerland

**Keywords:** crystal structure, hydro­boration, ynamine, push–pull olefin

## Abstract

*N*-[(*Z*)-2-(2*H*-1,3,2-Benzodioxaborol-2-yl)-2-phenyl­ethen­yl]-*N*-(propan-2-yl)aniline, C_23_H_22_BNO_2_, contains a C=C bond that is conjugated with a donor and an acceptor group. An analysis that included similar push–pull olefins revealed that bond lengths in their B—C=C—N core units correlate with the perceived acceptor and donor strength of the groups. The two phenyl groups in the mol­ecule are rotated with respect to the plane that contains the BCCN atoms, and are close enough for significant π-stacking. Definite characterization of the title compound demonstrates, for the first time in a reliable way, that hydro­boration of ynamines with borandiol esters is feasible. Compared to olefin hydro­boration with borane, the ynamine substrate is activated enough to undergo reaction with the less active hydro­boration reagent catecholborane.

## Abstract 2

The title compound is a demonstration of hydro­boration of ynamines with borandiol ester. The bond lengths of the resulting push–pull olefin are discussed.

## Chemical context   

Boronic esters are frequently used to transfer organic groups to transition metals, for example in the transmetallation step of the Suzuki–Miyaura reaction. Hydro­boration of ynamines with borandiol esters produces amino-functionalized boronic esters in one step and perfect atom economy.
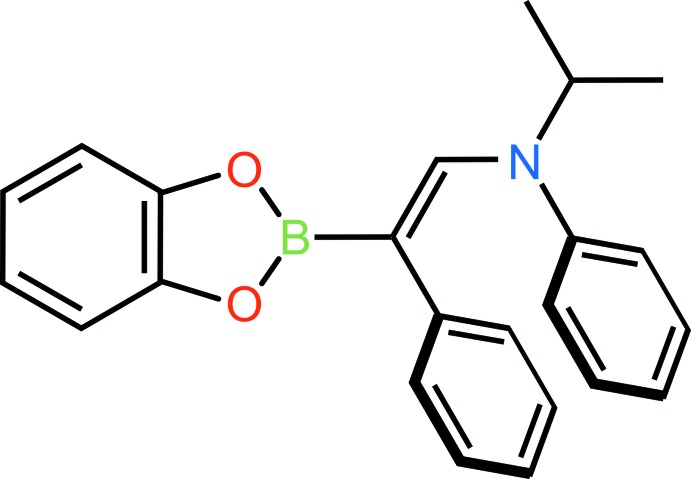


For true ynamines, to the best of our knowledge, only two attempts of such reactions have been reported so far. These either failed (Witulski *et al.*, 2000 ▸) or were reported without reaction details and characterization data (Zhuo *et al.*, 2001 ▸). More recently it was found that the exceptionally active Pier’s borane, HB(C_6_F_5_)_2_, can readily hydro­borate l-propynyl-2,2,6,6-tetra­methyl­piperidine (Wang *et al.*, 2018 ▸). Borandiol esters are expected to be less reactive because the electron deficiency at the boron is reduced by π-donation from the oxygen atoms.

Given the limited precedent for ynamine hydro­boration, the more comprehensive literature for enamine hydro­boration was consulted (Goralski & Singaram, 2012 ▸; Dembitsky *et al.*, 2002 ▸), as their reactivity is expected to be controlled by similar effects. Compared to simple olefin substrates, conjugation of the C=C bond with nitro­gen dictates the regioselectivity and increases the reactivity of enamines. However, the presence of a nitro­gen atom in the reactant and product enables the formation of unreactive Lewis acid–base adducts with the hydro­borating reagent. Building on the vast knowledge of the reactivity of different borane-amine adducts in hydro­boration (Brown & Murray, 1984 ▸; Brown *et al.*, 1999 ▸), a bulky *iso*-propyl and an phenyl group were selected as substituents for the ynamine nitro­gen. The former should weaken adducts for steric reasons, whereas the phenyl group is expected to reduce the nucleophilicity of the nitro­gen by conjugation.

Ynamine hydro­boration using a borandiol ester was reinvestigated and succeeded for a substrate that follows the developed design principles. The product of such a reaction contains a C=C double bond flanked by both an electron-donating group (EDG), the amine, and an electron-withdrawing group (EWG), the boronate. Therefore it belongs to the class of push–pull (captodative) olefins, which are known to have unusual properties such as weak π-bonds or biradical reactivity (Viehe *et al.*, 1985 ▸).

## Structural commentary   

**Figure 1:**
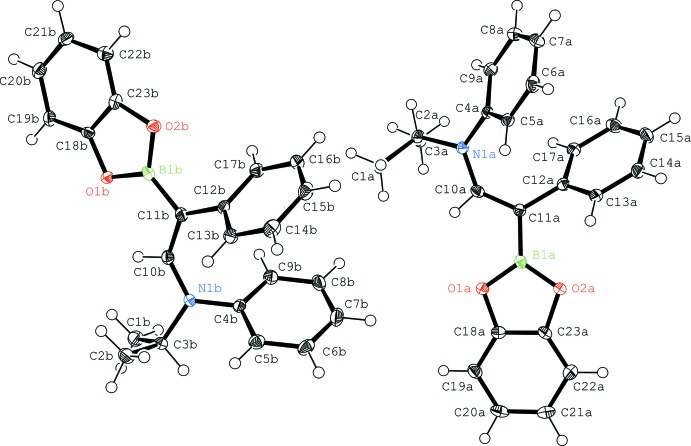
The mol­ecular structures of the two independent mol­ecules of the title compound **1** with displacement ellipsoids drawn at the 50% probability level.

The asymmetric unit (Fig. 1 ▸) contains two almost identical (r.m.d.s = 0.11 Å) independent mol­ecules. As judged by the B1—C11—C10—N1 torsion angles of 171.6 (2) and 175.5 (2)°, the central C—C bond is only slightly twisted from planarity. The two phenyl groups in the mol­ecule are rotated, by 43 and 49°, with respect to that plane. The centroids of two phenyl groups in one mol­ecule are on average 3.747 Å apart, which suggests intra­molecular π-stacking. The mean distances are 1.521 Å for the B1—C11 bond, 1.365 Å for the C10—N1 bond and 1.369 Å for the central C10—C11 bond.

## Supra­molecular features   

There is a short inter­molecular contact between the boron atom and an aniline *ortho*-H*B*1*B*⋯H9*A*(1 − *x*, 1 − *y*, 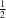 + *z*) = 2.771 Å and B1*A*⋯H5*B*(1 − *x*, −*y*, −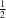 + *z*) = 2.856 Å (Σr_vdW_[B,H] = 3.11 Å). The shortest inter­molecular distances between the catechol unit and boron are B1*A*⋯C21*B* ^i^ = 3.540 (4) Å, B1*A*⋯C20*B* ^i^ = 3.686 (4) Å (Σr_vdW_[B,C] = 3.68 Å), B1*A*⋯H21*B* ^i^ = 3.113 Å and B1*A*⋯H20*B* ^i^ = 3.381 Å [symmetry code: (i) 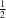 + *x*, 1 − *y*, *z*]. In addition there is a short contact between one of the other catechol hydrogen atoms and the *meta*-carbon of the aniline, H22*B*⋯C6*B*(*x*, 1 + *y*, *z*) of 2.877 Å (Σr_vdW_[C,H] = 2.97 Å). All of these inter­actions involve atoms that are part of arenes and could be seen as inter­molecular π-stacking.

Methyl hydrogen atoms of the isopropyl group are at van der Waals distances with one of the oxygen atoms [O2*A*⋯H2*BA*(1 − *x*, −*y*, −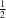 + *z*) = 2.698 Å, O2*B*⋯H3*AB*(1 − *x*, 1 − *y*, 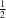 + *z*) = 2.631 Å, Σr_vdW_[O,H] = 2.70 Å] and with one of the anilic *meta*-H atoms [H8*A*⋯H2*BC*(1 − *x*, −*y*, −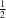 + *z*) = 2.388 Å, Σr_vdW_[H,H] = 2.40 Å]. The nitro­gen atom is steric­ally shielded by surrounding groups and does not have any close inter­molecular neighbours.

## Database survey   

**Figure 2:**
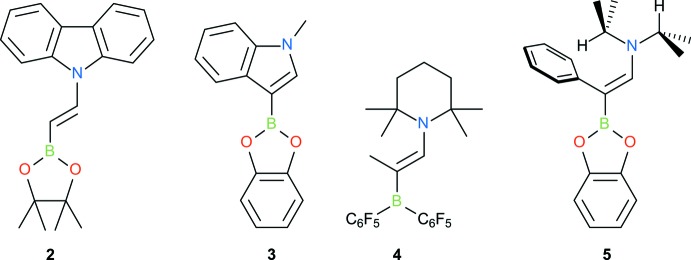
Chemical structure of reference compounds. 9-[(*E*)-2-(4,4,5,5-Tetra­methyl-1,3,2-dioxaborolan-2-yl)ethen­yl]-9*H*-carbazole, **2** (Hatayama & Okuno, 2012 ▸), 3-(2*H*-1,3,2-benzodioxaborol-2-yl)-1-methyl-1*H*-indole, **3** (Liu *et al.*, 2017 ▸), 1-{(*Z*)-2-[bis­(penta­fluoro­phen­yl)boran­yl]prop-1-en-1-yl}-2,2,6,6-tetra­methyl­piperidine, **4** (Wang *et al.*, 2018 ▸) and *N*-[(*Z*)-2-(2*H*-1,3,2-benzodioxaborol-2-yl)-2-phenyl­ethen­yl]-*N*-(propan-2-yl)propan-2-amine, **5** (CCDC 1997665).

Contributions from the zwitterionic resonance structure ^−^B=C—C=N^+^ are expected to increase with donor and acceptor group strength. This should be observable as a shortening of the B—C and C—N bonds and an elongation of the C—C bond. Following this idea, bond lengths of **1** were compared to those in the structurally related compounds **2**–**5** (Table 1 ▸, Fig. 2 ▸). C—N bond lengths are 1.341 Å (**4**), 1.350 Å (**5**), 1.362 Å (**3**), 1.365 Å (**1**), 1.394 Å (**2**). These numbers follow the expected N-donor strength, when the latter is estimated by the number of conjugating substituents on the nitro­gen: piperidine, diisopropyl > aniline > indole, carbazole. B—C lengths are 1.491 Å (**4**), 1.513 Å (**3**), 1.516 Å (**5**), 1.521 Å (**1**), 1.537 Å (**2**). Similarly, these numbers follow the B-acceptor strength: B(C_6_F_5_)_2_ > catecholboryl > pinacolboryl (Adamczyk-Woźniak *et al.*, 2011 ▸). Following this, the zwitterionic resonance structure is most important in **4**, which has a strong donor and a strong acceptor. On the other end of the scale lies **2**, which has a weak donor and a weak acceptor. The other mol­ecules, including **1**, lie between these two extremes.

**Figure 3:**
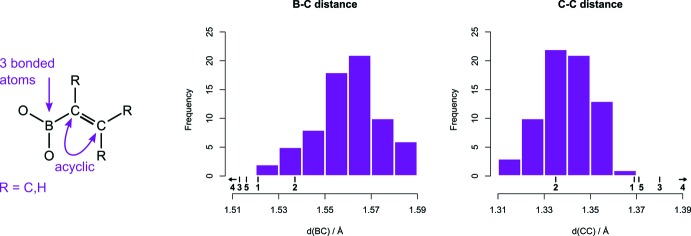
Statistical analysis of B—C and C—C bond lengths in vinyl boronates. The query substructure and restrictions are shown on the left. Problematic or irrelevant structures were removed. The bond distances of reference compounds are marked.

**Figure 4:**
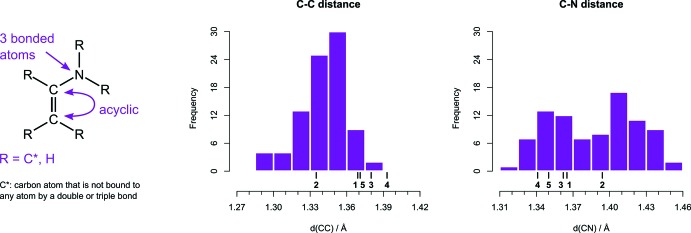
Statistical analysis of C—C and C—N bond lengths in enamines. The query substructure and restrictions are shown on the left. Problematic or irrelevant structures were removed. The bond distances of reference compounds are marked.

In order to compare with olefins that either have a donor or an acceptor group, the Cambridge Structural Database (CSD, Version 5.41, update of November 2019; Groom *et al.*, 2016 ▸) was searched for vinyl boronates and enamines. Bond-length distributions and the exact query structures are shown in Fig. 3 ▸ and Fig. 4 ▸. The data set for vinyl boronates consists of about 90% of pinacol boronates and contains only a few catechol boronates. Compared with typical bond lengths in this data set, the B—C bond is shorter and the C=C bond is longer in **1**–**5**, which indicates stronger conjugation. The only exception is **2**, whose C=C bond is shorter.

For enamines the C—C bond length has an average of 1.341 Å, which is about 0.025 Å longer than the value of 1.316 (15) Å for regular inter­nal olefins (Allen *et al.*, 2006 ▸). In **1**, **3**, **4** and **5**, the C—C bonds are on average 1.378 Å, and thereby longer than in enamines. C—N bond lengths for enamines are distributed more uniformly than C—C lengths. Inspection of the structures in which C—N distances are longer than 1.39 Å revealed that these structures typically either have a nitro­gen whose lone pair is not coplanar with the C=C bond, or a nitro­gen that is part of a carbazole or morpholine. As conjugation with the formal double bond between C10 and C11 is absent or reduced in these, only structures with C—N bond lengths below 1.39 Å were used for comparison. The average C—N bond length of about 1.36 Å for that subset is similar to the C—N bond lengths in **1**, **3**, **4** and **5**. Overall, comparison with enamines reveals that C—C bonds are longer in push–pull olefins, whereas C—N bond lengths are unaffected. This suggests that conjugation with the boron affects the C—C bond length more than the C—N bond length.

## Synthesis and crystallization   

**Figure 5:**
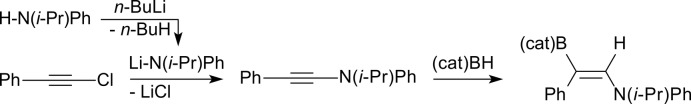
Reaction sequence used for the synthesis of the title compound.

The title compound was prepared by the multi-step sequence shown in Fig. 5 ▸.

***N*** **-isopropyl-** ***N*** **-(phenyl­ethyn­yl)aniline**: In a 100 ml Schlenk flask, 5.8 ml of *N*-isopropyl amine (40 mmol, 1.0 eq.) were diluted with 40 ml of dry THF. 24.7 ml of a *n*-BuLi solution in hexa­nes (1.62 mol l^−1^, 40 mmol, 1.0 eq.) were added over 5 min at 195 K. A colourless solid formed and after 15 min the suspension was warmed to room temperature over 30 min. Upon addition of 5.69 g of 2-chloro­ethynyl­benzene (96%, 40 mmol, 1.0 eq., prepared according to Li *et al.*, 2014 ▸), the reaction mixture turned black. The sealed Schlenk flask was heated in an oil bath at 338 K (caution: the closed flask may burst if this temperature is exceeded). The reaction progress was monitored by GC–FID. After 6 h the reaction mixture was cooled to room temperature. 100 ml of *tert*-butyl methyl ether were added, the organic phase washed with ice-cold water (3 × 50 ml), dried with MgSO_4_ and concentrated on the rotavap. 8.25 g of black viscous liquid were obtained and purified by Kugelrohr distillation (433 K, 0.2 mbar) to yield 6.34 g (purity 83 wt%, yield 56%) of the colourless liquid *N*-isopropyl-*N*-(phenyl­ethyn­yl)aniline. ^1^H NMR (400 MHz, CDCl_3_): δ (ppm) = 1.40 (*d*, 6.5 Hz, 6H, 2 × CH_3_), 4.10 (*hept*, 6.4 Hz, 1H, CH of *i*-Pr), 6.92 (*tt*, 7.4 Hz, 1.2 Hz, 1H, *para*-H of aniline), 7.16–7.33 (*m*, 7H, arene H), 7.38–7.41 (*m*, 2H, *ortho*-H of phenyl group). ^13^C{^1^H} NMR (100 MHz, CDCl_3_): δ (ppm) = 20.6 (*s*, 2 × CH_3_), 49.6 (*s*, CH of *i*-Pr), 72.0 (*s*, alkynic carbon farther from N), 86.4 (*s*, alkynic carbon closer to N), 115.5 (*s*, *ortho*-C of aniline), 120.8 (*s*, *para*-C of aniline), 125.2 (*s*, *ipso*-C of phenyl group), 126.2 (*s*, *para*-C of phenyl group), 128.4 (*s*, *meta*-C of phenyl group), 129.3 (*s*, *meta*-C of aniline), 130.2 (*s*, *ortho*-C of phenyl group), 144.4 (*s*, *ipso*-C of aniline). EI–MS (70 eV) *m*/*z* = 236, 235 (*M* ^+^), 220 (*M* ^+^ − CH_3_), 194, 193, 192 (*M* ^+^ − C_3_H_7_), 165, 117, 115, 90, 89, 77, 63, 51, 43. ATR–IR ν (cm^−1^)(%T) = 534 (70), 629 (61), 688 (22), 745 (18), 783 (82), 865 (87), 881 (87), 904 (87), 996 (80), 1025 (73), 1056 (66), 1129 (70), 1147 (52), 1170 (67), 1254 (41), 1312 (67), 1348 (77), 1367 (76), 1396 (51), 1490 (38), 1594 (46), 1932 (96), 2217 (38, C≡C-stretch), 2872 (94), 2932 (91), 2976 (80), 3034 (93), 3057 (93).

***N*** **-[(** ***Z*** **)-2-(2** ***H*** **-1,3,2-benzodioxaborol-2-yl)-2-phenyl­ethen­yl]-** ***N*** **-(propan-2-yl)aniline**: Under a counterflow of argon, 3.08 g of *N*-isopropyl-*N*-(phenyl­ethyn­yl)aniline (13.1 mmol, 1.0 eq.) were placed in an oven-dried 20 ml Schlenk flask with a Young valve. The flask and its contents were purged three times by applying high vacuum followed by flushing with argon. 6.5 ml dry *tert*-butyl methyl ether were added and the mixture stirred vigorously to ensure mixing of the two liquids. 2.1 ml of catecholborane (19.5 mmol, 1.5 eq.) were added, the flask closed, and the reaction mixture heated to 323 K for 16 h. Cooling to room temperature led to the precipitation of the product. The supernatant was removed and the precipitate recrystallized from 40 ml of *tert*-butyl methyl ether. X-ray quality crystals were obtained in a yield of 1.88 g (40%). Notes: (*a*) Schlenk techniques are necessary because ynamines and catecholborane are moisture-sensitive; (*b*) the reaction also works well in diethyl ether, 1,4-dioxane or THF.

^1^H NMR (600 MHz, CDCl_3_): δ (ppm) = 1.34 (*d*, 6.8 Hz, 6H, CH_3_ of *i*-Pr), 3.89 (*hept*, 6.8 Hz, 1H, CH of *i*-Pr), 6.77–6.80 (*m*, 2H, H5 & H9), 6.81–6.85 (*m*, 2H, H7 & H15), 6.85–6.92 (*m*, 6H, H6 & H8 & H13 & H14 & H16 & H17), 6.95–6.98 (*m*, 2H, 2 × catechol-H), 7.11–7.14 (*m*, 2H, 2 × catechol-H), 7.53 (*s*, 1H, H10).

^13^C^1^H NMR (151 MHz, CDCl_3_): δ (ppm) = 22.2 (*s*, C1 & C2), 57.4 (*s*, C3), 98.1 (*br s*, C11), 111.7 (*s*, 2 × catechol-C), 121.8 (*s*, 2 × catechol-C), 124.3 (*s*, C15), 124.6 (*s*, C7), 126.3 (*s*, C5 & C9), 126.9 (*s*, C14 & C16), 128.0 (*s*, C6 & C8), 129.4 (*s*, C13 & C17), 138.8 (*s*, C12), 143.3 (*s*, C4), 144.9 (*s*, C10), 148.9 (*s*, C18 & C23). Inverse gated {13}C{1}H} NMR with *D*1 = 60 s measured to get integrable ^13^C NMR.

^11^B NMR (160 MHz, CDCl_3_): δ (ppm) = 33.2 (*s*).

## Refinement   

Crystal data, data collection and structure refinement details are summarized in Table 2 ▸. H atoms were positioned geometrically and refined as riding: C—H = 0.95–0.98 Å and *U*_iso_(H) = 1.2*U*_eq_(C) or 1.5*U*_eq_(C-meth­yl). The absolute structure was not determined because of unreliable Flack and Hooft parameters.

## Supplementary Material

Crystal structure: contains datablock(s) I. DOI: 10.1107/S2056989020005289/lh5953sup1.cif

Structure factors: contains datablock(s) I. DOI: 10.1107/S2056989020005289/lh5953Isup2.hkl

Supporting information file. DOI: 10.1107/S2056989020005289/lh5953Isup3.cml

CCDC reference: 1997061

Additional supporting information:  crystallographic information; 3D view; checkCIF report

## Figures and Tables

**Table 1:** Comparison of bond lengths (in Å) in **1** with those in the similar compounds **2**–**5** Average distances and standard deviations are given whenever there is more than one mol­ecule in the asymmetric unit. Typical bond lengths for vinyl­boranes and conjugated enamines were obtained from statistical analysis.

Compound	B—C	C—C	C—N	CCDC
**1**	1.521 (3)	1.369 (3)	1.365 (3)	title compound
**2**	1.537 (4)	1.335 (4)	1.394 (3)	861787*^*a*^*
**3**	1.513 (4)	1.380 (3)	1.362 (3)	1529736
**4**	1.491 (7)	1.393 (6)	1.341 (6)	1843575^*c*^
**5**	1.516 (2)	1.371 (2)	1.350 (2)	1997665^*d*^
Vinyl boronates	1.561 (15)	1.341 (12)	–	–
Enamines	–	∼1.34	∼1.36	–

Notes: (*a*) Hatayama & Okuno (2012 ▸); (*b*) Liu *et al.* (2017 ▸); (*c*) Wang *et al.* (2018 ▸); (*d*) new.

**Table 2:** Experimental details

Crystal data	
Chemical formula	C_23_H_22_BNO_2_
*M*_r_	355.22
Crystal system, space group	Orthorhombic, *P* *c* *a*2_1_
Temperature (K)	100
*a*, *b*, *c* (Å)	17.8540 (11), 11.5361 (6), 18.5366 (12)
*V* (Å^3^)	3817.9 (4)
*Z*	8
Radiation type	Mo *K*α
μ (mm^−1^)	0.08
Crystal size (mm)	0.38 × 0.2 × 0.07

Data collection	
Diffractometer	Bruker–Nonius Kappa APEXII
Absorption correction	Multi-scan (*SADABS*; Bruker, 2015 ▸)
*T*_min_, *T*_max_	0.703, 0.733
No. of measured, independent and observed [*I* > 2σ(*I*)] reflections	60829, 8781, 7379
*R*_int_	0.047
(sin θ/λ)_max_ (Å^−1^)	0.652

Refinement	
*R*[*F* ^2^ > 2σ(*F* ^2^)], *wR*(*F* ^2^), *S*	0.039, 0.086, 1.03
No. of reflections	8781
No. of parameters	491
No. of restraints	1
H-atom treatment	H-atom parameters constrained
Δρ_max_, Δρ_min_ (e Å^−3^)	0.25, −0.24

Computer programs: *APEX2* and *SAINT* (Bruker, 2015 ▸), *SHELXT* (Sheldrick, 2015*a* ▸), *SHELXL* (Sheldrick, 2015*b* ▸) and *OLEX2* (Dolomanov *et al.*, 2009 ▸).

## supplementary crystallographic information

### Crystal data

**Table:**

C_23_H_22_BNO_2_	*D*_x_ = 1.236 Mg m^−^^3^
*M**_r_* = 355.22	Mo *K*α radiation, λ = 0.71073 Å
Orthorhombic, *P**c**a*2_1_	Cell parameters from 9981 reflections
*a* = 17.8540 (11) Å	θ = 2.5–26.8°
*b* = 11.5361 (6) Å	µ = 0.08 mm^−^^1^
*c* = 18.5366 (12) Å	*T* = 100 K
*V* = 3817.9 (4) Å^3^	Plate, clear light yellow
*Z* = 8	0.38 × 0.2 × 0.07 mm
*F*(000) = 1504	

### Data collection

**Table:**

Bruker–Nonius Kappa APEXII diffractometer	8781 independent reflections
Radiation source: sealed tube	7379 reflections with *I* > 2σ(*I*)
Graphite monochromator	*R*_int_ = 0.047
Detector resolution: 8.33 pixels mm^-1^	θ_max_ = 27.6°, θ_min_ = 2.8°
ω and φ scans	*h* = −23→23
Absorption correction: multi-scan (SADABS; Bruker, 2015)	*k* = −14→14
*T*_min_ = 0.703, *T*_max_ = 0.733	*l* = −24→24
60829 measured reflections	

### Refinement

**Table:**

Refinement on *F*^2^	Hydrogen site location: inferred from neighbouring sites
Least-squares matrix: full	H-atom parameters constrained
*R*[*F*^2^ > 2σ(*F*^2^)] = 0.039	*w* = 1/[σ^2^(*F*_o_^2^) + (0.0401*P*)^2^ + 0.7053*P*] where *P* = (*F*_o_^2^ + 2*F*_c_^2^)/3
*wR*(*F*^2^) = 0.086	(Δ/σ)_max_ < 0.001
*S* = 1.03	Δρ_max_ = 0.25 e Å^−^^3^
8781 reflections	Δρ_min_ = −0.24 e Å^−^^3^
491 parameters	Absolute structure: Flack x determined using 3185 quotients [(I+)-(I-)]/[(I+)+(I-)] (Parsons, Flack and Wagner, Acta Cryst. B69 (2013) 249-259).
1 restraint	Absolute structure parameter: −0.7 (4)
Primary atom site location: structure-invariant direct methods	

### Special details

**Table:**

Geometry. All esds (except the esd in the dihedral angle between two l.s. planes) are estimated using the full covariance matrix. The cell esds are taken into account individually in the estimation of esds in distances, angles and torsion angles; correlations between esds in cell parameters are only used when they are defined by crystal symmetry. An approximate (isotropic) treatment of cell esds is used for estimating esds involving l.s. planes.
Refinement. 8 reflections were omitted (some are equivalents). These were checked visually and are all results of high background around the beamstop (beginning ice formation or crystalline powder covering the sample). 0 1 0 is clearly shadowed by the beamstop. Absolute structure is not claimed due to unreliable Flack and Hooft parameters.

### Fractional atomic coordinates and isotropic or equivalent isotropic displacement parameters (Å^2^)

**Table:**

	*x*	*y*	*z*	*U*_iso_*/*U*_eq_	
O1B	0.18188 (9)	0.59842 (13)	0.60028 (9)	0.0155 (4)	
O2B	0.30086 (9)	0.67252 (13)	0.61169 (9)	0.0158 (4)	
N1B	0.23745 (11)	0.25154 (16)	0.65127 (11)	0.0159 (4)	
C1B	0.10503 (15)	0.2002 (2)	0.62440 (14)	0.0210 (6)	
H1BA	0.0874	0.2806	0.6251	0.032*	
H1BB	0.0642	0.1485	0.6390	0.032*	
H1BC	0.1215	0.1800	0.5756	0.032*	
C2B	0.14877 (15)	0.2209 (2)	0.75303 (14)	0.0205 (6)	
H2BA	0.1928	0.2152	0.7844	0.031*	
H2BB	0.1096	0.1686	0.7707	0.031*	
H2BC	0.1301	0.3008	0.7533	0.031*	
C3B	0.17032 (14)	0.1869 (2)	0.67659 (13)	0.0160 (5)	
H3B	0.1840	0.1028	0.6776	0.019*	
C4B	0.30570 (13)	0.18933 (19)	0.64199 (14)	0.0147 (5)	
C5B	0.32986 (14)	0.1121 (2)	0.69452 (14)	0.0189 (5)	
H5B	0.3009	0.1007	0.7369	0.023*	
C6B	0.39606 (14)	0.0518 (2)	0.68521 (16)	0.0230 (6)	
H6B	0.4126	−0.0006	0.7214	0.028*	
C7B	0.43822 (15)	0.0673 (2)	0.62365 (16)	0.0275 (6)	
H7B	0.4841	0.0267	0.6177	0.033*	
C8B	0.41341 (15)	0.1423 (2)	0.57066 (15)	0.0238 (6)	
H8B	0.4420	0.1522	0.5279	0.029*	
C9B	0.34704 (14)	0.2033 (2)	0.57938 (14)	0.0191 (5)	
H9B	0.3301	0.2543	0.5426	0.023*	
C10B	0.23179 (13)	0.36795 (19)	0.63924 (13)	0.0151 (5)	
H10B	0.1820	0.3949	0.6321	0.018*	
C11B	0.28538 (13)	0.4526 (2)	0.63563 (13)	0.0156 (5)	
C12B	0.36555 (13)	0.4370 (2)	0.65557 (13)	0.0142 (5)	
C13B	0.38562 (14)	0.3878 (2)	0.72178 (13)	0.0172 (5)	
H13B	0.3476	0.3630	0.7541	0.021*	
C14B	0.46041 (15)	0.3748 (2)	0.74087 (14)	0.0209 (5)	
H14B	0.4731	0.3410	0.7860	0.025*	
C15B	0.51658 (14)	0.4110 (2)	0.69433 (14)	0.0210 (6)	
H15B	0.5677	0.4012	0.7071	0.025*	
C16B	0.49740 (14)	0.4614 (2)	0.62906 (14)	0.0188 (5)	
H16B	0.5356	0.4869	0.5972	0.023*	
C17B	0.42288 (14)	0.4749 (2)	0.60995 (14)	0.0169 (5)	
H17B	0.4106	0.5103	0.5652	0.020*	
C18B	0.18063 (14)	0.71629 (19)	0.58605 (13)	0.0140 (5)	
C19B	0.12056 (14)	0.7829 (2)	0.56544 (13)	0.0178 (5)	
H19B	0.0715	0.7518	0.5614	0.021*	
C20B	0.13606 (15)	0.8997 (2)	0.55079 (14)	0.0198 (5)	
H20B	0.0966	0.9492	0.5356	0.024*	
C21B	0.20801 (15)	0.9445 (2)	0.55794 (13)	0.0192 (5)	
H21B	0.2165	1.0242	0.5478	0.023*	
C22B	0.26793 (14)	0.8754 (2)	0.57958 (13)	0.0179 (5)	
H22B	0.3171	0.9059	0.5849	0.022*	
C23B	0.25211 (13)	0.76087 (19)	0.59287 (13)	0.0151 (5)	
B1B	0.25681 (15)	0.5724 (2)	0.61526 (14)	0.0149 (6)	
O1A	0.59813 (9)	−0.09865 (13)	0.40292 (10)	0.0176 (4)	
O2A	0.71747 (9)	−0.17253 (13)	0.39845 (9)	0.0174 (4)	
N1A	0.65302 (11)	0.24387 (17)	0.34366 (11)	0.0161 (4)	
C1A	0.52189 (14)	0.2942 (2)	0.37802 (15)	0.0228 (6)	
H1AA	0.5416	0.3104	0.4263	0.034*	
H1AB	0.5027	0.2146	0.3764	0.034*	
H1AC	0.4813	0.3485	0.3671	0.034*	
C2A	0.58421 (14)	0.3081 (2)	0.32256 (14)	0.0177 (5)	
H2A	0.5976	0.3923	0.3213	0.021*	
C3A	0.55869 (14)	0.2754 (2)	0.24731 (15)	0.0227 (6)	
H3AA	0.5407	0.1952	0.2473	0.034*	
H3AB	0.6008	0.2829	0.2137	0.034*	
H3AC	0.5180	0.3272	0.2323	0.034*	
C4A	0.72025 (13)	0.3083 (2)	0.35237 (13)	0.0139 (5)	
C5A	0.76423 (13)	0.2910 (2)	0.41332 (13)	0.0150 (5)	
H5A	0.7496	0.2358	0.4487	0.018*	
C6A	0.82952 (14)	0.3543 (2)	0.42245 (14)	0.0199 (5)	
H6A	0.8596	0.3423	0.4640	0.024*	
C7A	0.85112 (15)	0.4353 (2)	0.37098 (15)	0.0233 (6)	
H7A	0.8964	0.4776	0.3768	0.028*	
C8A	0.80630 (14)	0.4541 (2)	0.31124 (16)	0.0227 (6)	
H8A	0.8204	0.5105	0.2765	0.027*	
C9A	0.74113 (14)	0.3912 (2)	0.30178 (14)	0.0182 (5)	
H9A	0.7106	0.4047	0.2607	0.022*	
C10A	0.64818 (14)	0.1276 (2)	0.35658 (13)	0.0157 (5)	
H10A	0.5984	0.1001	0.3624	0.019*	
C11A	0.70180 (13)	0.0433 (2)	0.36266 (12)	0.0136 (5)	
C12A	0.78275 (13)	0.0582 (2)	0.34518 (13)	0.0143 (5)	
C13A	0.83799 (13)	0.0207 (2)	0.39292 (13)	0.0159 (5)	
H13A	0.8239	−0.0154	0.4369	0.019*	
C14A	0.91359 (14)	0.0353 (2)	0.37700 (15)	0.0184 (5)	
H14A	0.9505	0.0104	0.4105	0.022*	
C15A	0.93517 (15)	0.0860 (2)	0.31277 (14)	0.0209 (6)	
H15A	0.9868	0.0967	0.3021	0.025*	
C16A	0.88076 (14)	0.1212 (2)	0.26390 (14)	0.0209 (6)	
H16A	0.8952	0.1549	0.2193	0.025*	
C17A	0.80555 (14)	0.1074 (2)	0.27994 (13)	0.0166 (5)	
H17A	0.7689	0.1317	0.2460	0.020*	
C18A	0.59638 (14)	−0.2147 (2)	0.42114 (13)	0.0155 (5)	
C19A	0.53579 (14)	−0.2820 (2)	0.43916 (14)	0.0196 (5)	
H19A	0.4864	−0.2514	0.4402	0.024*	
C20A	0.55064 (15)	−0.3974 (2)	0.45574 (14)	0.0212 (6)	
H20A	0.5102	−0.4469	0.4685	0.025*	
C21A	0.62240 (15)	−0.4420 (2)	0.45425 (14)	0.0221 (6)	
H21A	0.6302	−0.5212	0.4661	0.027*	
C22A	0.68384 (15)	−0.3727 (2)	0.43561 (14)	0.0215 (6)	
H22A	0.7335	−0.4024	0.4346	0.026*	
C23A	0.66814 (14)	−0.2591 (2)	0.41882 (13)	0.0159 (5)	
B1A	0.67343 (15)	−0.0740 (2)	0.38806 (15)	0.0149 (5)	

### Atomic displacement parameters (Å^2^)

**Table:**

	*U*^11^	*U*^22^	*U*^33^	*U*^12^	*U*^13^	*U*^23^
O1B	0.0139 (8)	0.0119 (8)	0.0206 (9)	0.0005 (7)	0.0001 (7)	0.0023 (7)
O2B	0.0137 (8)	0.0144 (8)	0.0194 (9)	−0.0010 (6)	−0.0018 (7)	0.0016 (7)
N1B	0.0115 (10)	0.0142 (9)	0.0218 (11)	0.0004 (8)	0.0030 (8)	0.0028 (8)
C1B	0.0183 (13)	0.0203 (12)	0.0245 (15)	−0.0017 (10)	−0.0016 (11)	−0.0009 (11)
C2B	0.0194 (14)	0.0215 (13)	0.0206 (13)	−0.0021 (11)	0.0031 (11)	0.0012 (10)
C3B	0.0142 (13)	0.0135 (11)	0.0205 (13)	−0.0017 (10)	0.0030 (10)	0.0013 (10)
C4B	0.0115 (12)	0.0112 (10)	0.0213 (13)	−0.0010 (9)	−0.0012 (10)	−0.0023 (10)
C5B	0.0187 (13)	0.0162 (12)	0.0218 (14)	−0.0021 (10)	−0.0029 (10)	0.0006 (10)
C6B	0.0174 (13)	0.0157 (12)	0.0360 (16)	0.0007 (10)	−0.0082 (12)	0.0023 (11)
C7B	0.0144 (13)	0.0196 (12)	0.0487 (19)	0.0027 (11)	0.0015 (13)	−0.0046 (12)
C8B	0.0196 (14)	0.0191 (13)	0.0328 (16)	−0.0020 (11)	0.0108 (11)	−0.0065 (11)
C9B	0.0173 (13)	0.0168 (12)	0.0232 (13)	−0.0015 (10)	0.0015 (10)	−0.0014 (10)
C10B	0.0132 (12)	0.0174 (11)	0.0147 (11)	0.0022 (9)	0.0007 (9)	0.0013 (10)
C11B	0.0146 (12)	0.0169 (11)	0.0152 (12)	0.0014 (10)	0.0027 (10)	0.0006 (10)
C12B	0.0133 (12)	0.0120 (11)	0.0173 (12)	0.0018 (9)	0.0002 (10)	−0.0012 (10)
C13B	0.0182 (13)	0.0161 (12)	0.0172 (13)	0.0000 (10)	0.0021 (10)	−0.0014 (10)
C14B	0.0217 (14)	0.0217 (13)	0.0195 (13)	0.0023 (11)	−0.0053 (11)	0.0007 (11)
C15B	0.0143 (13)	0.0208 (12)	0.0281 (15)	0.0023 (10)	−0.0057 (11)	−0.0022 (11)
C16B	0.0144 (12)	0.0178 (12)	0.0244 (14)	−0.0007 (10)	0.0032 (10)	−0.0005 (10)
C17B	0.0177 (12)	0.0163 (11)	0.0169 (12)	0.0007 (10)	0.0008 (10)	0.0008 (10)
C18B	0.0171 (12)	0.0124 (11)	0.0124 (11)	−0.0024 (10)	0.0031 (9)	0.0000 (9)
C19B	0.0140 (13)	0.0202 (13)	0.0191 (13)	0.0031 (10)	0.0011 (10)	0.0001 (10)
C20B	0.0236 (14)	0.0186 (12)	0.0171 (13)	0.0061 (11)	0.0014 (11)	0.0014 (10)
C21B	0.0277 (14)	0.0125 (11)	0.0172 (13)	0.0003 (11)	0.0044 (11)	−0.0002 (10)
C22B	0.0217 (13)	0.0174 (11)	0.0147 (12)	−0.0032 (10)	0.0000 (10)	−0.0025 (9)
C23B	0.0158 (12)	0.0167 (11)	0.0127 (11)	0.0028 (10)	−0.0025 (9)	−0.0023 (10)
B1B	0.0128 (13)	0.0191 (13)	0.0129 (13)	−0.0004 (11)	0.0016 (11)	−0.0006 (11)
O1A	0.0134 (9)	0.0138 (8)	0.0258 (10)	−0.0012 (7)	0.0003 (7)	0.0027 (7)
O2A	0.0139 (9)	0.0146 (8)	0.0237 (10)	−0.0005 (7)	0.0028 (7)	0.0023 (7)
N1A	0.0116 (10)	0.0147 (10)	0.0220 (11)	0.0002 (8)	−0.0036 (8)	0.0030 (8)
C1A	0.0161 (13)	0.0221 (13)	0.0304 (15)	0.0042 (11)	0.0001 (11)	0.0005 (12)
C2A	0.0128 (12)	0.0150 (11)	0.0251 (14)	0.0015 (10)	−0.0032 (10)	0.0033 (10)
C3A	0.0154 (14)	0.0273 (14)	0.0254 (14)	0.0027 (11)	−0.0061 (11)	0.0047 (11)
C4A	0.0121 (12)	0.0119 (11)	0.0175 (12)	0.0009 (9)	0.0005 (10)	−0.0018 (9)
C5A	0.0157 (12)	0.0142 (10)	0.0151 (12)	0.0010 (9)	0.0010 (10)	−0.0004 (9)
C6A	0.0160 (12)	0.0214 (12)	0.0223 (14)	0.0021 (10)	−0.0038 (10)	−0.0035 (11)
C7A	0.0155 (13)	0.0179 (12)	0.0366 (16)	−0.0054 (11)	0.0001 (12)	−0.0006 (11)
C8A	0.0198 (14)	0.0182 (12)	0.0300 (15)	−0.0014 (11)	0.0034 (11)	0.0066 (11)
C9A	0.0175 (13)	0.0173 (12)	0.0198 (13)	0.0018 (10)	−0.0009 (10)	0.0033 (10)
C10A	0.0129 (12)	0.0171 (12)	0.0172 (12)	−0.0036 (10)	−0.0012 (9)	0.0010 (10)
C11A	0.0125 (12)	0.0146 (11)	0.0138 (12)	0.0000 (9)	−0.0014 (9)	0.0005 (9)
C12A	0.0143 (12)	0.0123 (11)	0.0164 (12)	−0.0009 (9)	0.0007 (10)	−0.0042 (10)
C13A	0.0162 (12)	0.0143 (11)	0.0170 (13)	0.0002 (10)	0.0011 (10)	0.0002 (10)
C14A	0.0140 (12)	0.0166 (12)	0.0247 (14)	0.0000 (10)	−0.0032 (10)	−0.0033 (10)
C15A	0.0152 (13)	0.0220 (12)	0.0255 (14)	−0.0017 (10)	0.0056 (11)	−0.0028 (11)
C16A	0.0229 (14)	0.0215 (13)	0.0182 (13)	−0.0034 (11)	0.0064 (11)	0.0000 (11)
C17A	0.0165 (12)	0.0167 (12)	0.0166 (12)	−0.0003 (10)	−0.0014 (10)	−0.0008 (10)
C18A	0.0185 (13)	0.0154 (11)	0.0127 (12)	−0.0018 (10)	−0.0012 (9)	0.0004 (9)
C19A	0.0151 (13)	0.0228 (13)	0.0211 (13)	−0.0022 (11)	−0.0002 (10)	0.0028 (11)
C20A	0.0236 (14)	0.0209 (13)	0.0190 (13)	−0.0103 (11)	−0.0021 (11)	0.0036 (10)
C21A	0.0294 (15)	0.0155 (12)	0.0215 (13)	−0.0025 (11)	−0.0005 (11)	0.0047 (10)
C22A	0.0216 (14)	0.0191 (12)	0.0237 (14)	0.0030 (11)	−0.0002 (11)	0.0019 (11)
C23A	0.0137 (12)	0.0178 (12)	0.0164 (12)	−0.0037 (10)	0.0009 (10)	0.0009 (10)
B1A	0.0126 (13)	0.0177 (13)	0.0143 (13)	−0.0006 (11)	0.0002 (11)	−0.0026 (11)

### Geometric parameters (Å, º)

**Table:**

O1B—C18B	1.385 (3)	O1A—C18A	1.382 (3)
O1B—B1B	1.399 (3)	O1A—B1A	1.402 (3)
O2B—C23B	1.385 (3)	O2A—C23A	1.384 (3)
O2B—B1B	1.398 (3)	O2A—B1A	1.396 (3)
N1B—C3B	1.488 (3)	N1A—C2A	1.487 (3)
N1B—C4B	1.425 (3)	N1A—C4A	1.421 (3)
N1B—C10B	1.365 (3)	N1A—C10A	1.365 (3)
C1B—H1BA	0.9800	C1A—H1AA	0.9800
C1B—H1BB	0.9800	C1A—H1AB	0.9800
C1B—H1BC	0.9800	C1A—H1AC	0.9800
C1B—C3B	1.523 (4)	C1A—C2A	1.523 (4)
C2B—H2BA	0.9800	C2A—H2A	1.0000
C2B—H2BB	0.9800	C2A—C3A	1.515 (4)
C2B—H2BC	0.9800	C3A—H3AA	0.9800
C2B—C3B	1.520 (3)	C3A—H3AB	0.9800
C3B—H3B	1.0000	C3A—H3AC	0.9800
C4B—C5B	1.389 (3)	C4A—C5A	1.390 (3)
C4B—C9B	1.385 (4)	C4A—C9A	1.390 (3)
C5B—H5B	0.9500	C5A—H5A	0.9500
C5B—C6B	1.382 (3)	C5A—C6A	1.386 (3)
C6B—H6B	0.9500	C6A—H6A	0.9500
C6B—C7B	1.379 (4)	C6A—C7A	1.390 (4)
C7B—H7B	0.9500	C7A—H7A	0.9500
C7B—C8B	1.382 (4)	C7A—C8A	1.383 (4)
C8B—H8B	0.9500	C8A—H8A	0.9500
C8B—C9B	1.387 (4)	C8A—C9A	1.382 (4)
C9B—H9B	0.9500	C9A—H9A	0.9500
C10B—H10B	0.9500	C10A—H10A	0.9500
C10B—C11B	1.369 (3)	C10A—C11A	1.370 (3)
C11B—C12B	1.489 (3)	C11A—C12A	1.491 (3)
C11B—B1B	1.521 (4)	C11A—B1A	1.519 (3)
C12B—C13B	1.399 (3)	C12A—C13A	1.394 (3)
C12B—C17B	1.398 (3)	C12A—C17A	1.397 (3)
C13B—H13B	0.9500	C13A—H13A	0.9500
C13B—C14B	1.389 (4)	C13A—C14A	1.392 (3)
C14B—H14B	0.9500	C14A—H14A	0.9500
C14B—C15B	1.387 (4)	C14A—C15A	1.381 (4)
C15B—H15B	0.9500	C15A—H15A	0.9500
C15B—C16B	1.385 (4)	C15A—C16A	1.389 (4)
C16B—H16B	0.9500	C16A—H16A	0.9500
C16B—C17B	1.386 (3)	C16A—C17A	1.385 (3)
C17B—H17B	0.9500	C17A—H17A	0.9500
C18B—C19B	1.374 (3)	C18A—C19A	1.372 (3)
C18B—C23B	1.382 (3)	C18A—C23A	1.381 (3)
C19B—H19B	0.9500	C19A—H19A	0.9500
C19B—C20B	1.401 (3)	C20A—H20A	0.9500
C20B—H20B	0.9500	C20A—C21A	1.381 (4)
C20B—C21B	1.391 (4)	C21A—H21A	0.9500
C21B—H21B	0.9500	C21A—C22A	1.401 (4)
C21B—C22B	1.393 (4)	C22A—H22A	0.9500
C22B—H22B	0.9500	C22A—C23A	1.375 (3)
C22B—C23B	1.374 (3)		
			
C18B—O1B—B1B	105.28 (18)	C18A—O1A—B1A	105.46 (18)
C23B—O2B—B1B	105.43 (18)	C23A—O2A—B1A	105.50 (18)
C4B—N1B—C3B	118.34 (18)	C4A—N1A—C2A	117.83 (18)
C10B—N1B—C3B	119.02 (19)	C10A—N1A—C2A	118.9 (2)
C10B—N1B—C4B	122.6 (2)	C10A—N1A—C4A	123.2 (2)
H1BA—C1B—H1BB	109.5	H1AA—C1A—H1AB	109.5
H1BA—C1B—H1BC	109.5	H1AA—C1A—H1AC	109.5
H1BB—C1B—H1BC	109.5	H1AB—C1A—H1AC	109.5
C3B—C1B—H1BA	109.5	C2A—C1A—H1AA	109.5
C3B—C1B—H1BB	109.5	C2A—C1A—H1AB	109.5
C3B—C1B—H1BC	109.5	C2A—C1A—H1AC	109.5
H2BA—C2B—H2BB	109.5	N1A—C2A—C1A	111.9 (2)
H2BA—C2B—H2BC	109.5	N1A—C2A—H2A	107.0
H2BB—C2B—H2BC	109.5	N1A—C2A—C3A	111.5 (2)
C3B—C2B—H2BA	109.5	C1A—C2A—H2A	107.0
C3B—C2B—H2BB	109.5	C3A—C2A—C1A	112.1 (2)
C3B—C2B—H2BC	109.5	C3A—C2A—H2A	107.0
N1B—C3B—C1B	111.5 (2)	C2A—C3A—H3AA	109.5
N1B—C3B—C2B	111.6 (2)	C2A—C3A—H3AB	109.5
N1B—C3B—H3B	107.2	C2A—C3A—H3AC	109.5
C1B—C3B—H3B	107.2	H3AA—C3A—H3AB	109.5
C2B—C3B—C1B	111.9 (2)	H3AA—C3A—H3AC	109.5
C2B—C3B—H3B	107.2	H3AB—C3A—H3AC	109.5
C5B—C4B—N1B	120.3 (2)	C5A—C4A—N1A	119.6 (2)
C9B—C4B—N1B	119.9 (2)	C5A—C4A—C9A	119.7 (2)
C9B—C4B—C5B	119.8 (2)	C9A—C4A—N1A	120.6 (2)
C4B—C5B—H5B	120.0	C4A—C5A—H5A	120.1
C6B—C5B—C4B	120.1 (2)	C6A—C5A—C4A	119.9 (2)
C6B—C5B—H5B	120.0	C6A—C5A—H5A	120.1
C5B—C6B—H6B	119.8	C5A—C6A—H6A	119.9
C7B—C6B—C5B	120.3 (2)	C5A—C6A—C7A	120.3 (2)
C7B—C6B—H6B	119.8	C7A—C6A—H6A	119.9
C6B—C7B—H7B	120.2	C6A—C7A—H7A	120.2
C6B—C7B—C8B	119.7 (2)	C8A—C7A—C6A	119.6 (2)
C8B—C7B—H7B	120.2	C8A—C7A—H7A	120.2
C7B—C8B—H8B	119.7	C7A—C8A—H8A	119.8
C7B—C8B—C9B	120.5 (2)	C9A—C8A—C7A	120.4 (2)
C9B—C8B—H8B	119.7	C9A—C8A—H8A	119.8
C4B—C9B—C8B	119.6 (2)	C4A—C9A—H9A	120.0
C4B—C9B—H9B	120.2	C8A—C9A—C4A	120.1 (2)
C8B—C9B—H9B	120.2	C8A—C9A—H9A	120.0
N1B—C10B—H10B	114.4	N1A—C10A—H10A	114.0
N1B—C10B—C11B	131.2 (2)	N1A—C10A—C11A	131.9 (2)
C11B—C10B—H10B	114.4	C11A—C10A—H10A	114.0
C10B—C11B—C12B	125.0 (2)	C10A—C11A—C12A	125.3 (2)
C10B—C11B—B1B	115.2 (2)	C10A—C11A—B1A	115.1 (2)
C12B—C11B—B1B	119.6 (2)	C12A—C11A—B1A	119.5 (2)
C13B—C12B—C11B	120.9 (2)	C13A—C12A—C11A	120.8 (2)
C17B—C12B—C11B	121.1 (2)	C13A—C12A—C17A	118.0 (2)
C17B—C12B—C13B	118.0 (2)	C17A—C12A—C11A	121.2 (2)
C12B—C13B—H13B	119.6	C12A—C13A—H13A	119.5
C14B—C13B—C12B	120.9 (2)	C14A—C13A—C12A	120.9 (2)
C14B—C13B—H13B	119.6	C14A—C13A—H13A	119.5
C13B—C14B—H14B	119.9	C13A—C14A—H14A	119.8
C15B—C14B—C13B	120.3 (2)	C15A—C14A—C13A	120.3 (2)
C15B—C14B—H14B	119.9	C15A—C14A—H14A	119.8
C14B—C15B—H15B	120.3	C14A—C15A—H15A	120.3
C16B—C15B—C14B	119.4 (2)	C14A—C15A—C16A	119.4 (2)
C16B—C15B—H15B	120.3	C16A—C15A—H15A	120.3
C15B—C16B—H16B	119.7	C15A—C16A—H16A	119.9
C15B—C16B—C17B	120.5 (2)	C17A—C16A—C15A	120.3 (2)
C17B—C16B—H16B	119.7	C17A—C16A—H16A	119.9
C12B—C17B—H17B	119.5	C12A—C17A—H17A	119.5
C16B—C17B—C12B	120.9 (2)	C16A—C17A—C12A	121.0 (2)
C16B—C17B—H17B	119.5	C16A—C17A—H17A	119.5
C19B—C18B—O1B	127.9 (2)	C19A—C18A—O1A	128.7 (2)
C19B—C18B—C23B	122.6 (2)	C19A—C18A—C23A	122.0 (2)
C23B—C18B—O1B	109.4 (2)	C23A—C18A—O1A	109.3 (2)
C18B—C19B—H19B	122.0	C18A—C19A—H19A	121.8
C18B—C19B—C20B	115.9 (2)	C18A—C19A—C20A	116.4 (2)
C20B—C19B—H19B	122.0	C20A—C19A—H19A	121.8
C19B—C20B—H20B	119.3	C19A—C20A—H20A	119.0
C21B—C20B—C19B	121.4 (2)	C21A—C20A—C19A	121.9 (2)
C21B—C20B—H20B	119.3	C21A—C20A—H20A	119.0
C20B—C21B—H21B	119.2	C20A—C21A—H21A	119.4
C20B—C21B—C22B	121.6 (2)	C20A—C21A—C22A	121.3 (2)
C22B—C21B—H21B	119.2	C22A—C21A—H21A	119.4
C21B—C22B—H22B	121.8	C21A—C22A—H22A	122.0
C23B—C22B—C21B	116.4 (2)	C23A—C22A—C21A	116.1 (2)
C23B—C22B—H22B	121.8	C23A—C22A—H22A	122.0
C18B—C23B—O2B	109.24 (19)	C18A—C23A—O2A	109.3 (2)
C22B—C23B—O2B	128.6 (2)	C22A—C23A—O2A	128.3 (2)
C22B—C23B—C18B	122.1 (2)	C22A—C23A—C18A	122.4 (2)
O1B—B1B—C11B	124.4 (2)	O1A—B1A—C11A	124.2 (2)
O2B—B1B—O1B	110.6 (2)	O2A—B1A—O1A	110.4 (2)
O2B—B1B—C11B	125.0 (2)	O2A—B1A—C11A	125.5 (2)
			
O1B—C18B—C19B—C20B	−176.7 (2)	O1A—C18A—C19A—C20A	−179.5 (2)
O1B—C18B—C23B—O2B	0.0 (3)	O1A—C18A—C23A—O2A	−0.5 (3)
O1B—C18B—C23B—C22B	177.9 (2)	O1A—C18A—C23A—C22A	178.9 (2)
N1B—C4B—C5B—C6B	179.9 (2)	N1A—C4A—C5A—C6A	−179.4 (2)
N1B—C4B—C9B—C8B	−179.9 (2)	N1A—C4A—C9A—C8A	179.4 (2)
N1B—C10B—C11B—C12B	−9.7 (4)	N1A—C10A—C11A—C12A	−9.7 (4)
N1B—C10B—C11B—B1B	175.5 (2)	N1A—C10A—C11A—B1A	171.6 (2)
C3B—N1B—C4B—C5B	−45.6 (3)	C2A—N1A—C4A—C5A	132.7 (2)
C3B—N1B—C4B—C9B	132.5 (2)	C2A—N1A—C4A—C9A	−45.1 (3)
C3B—N1B—C10B—C11B	159.6 (3)	C2A—N1A—C10A—C11A	165.3 (2)
C4B—N1B—C3B—C1B	−123.7 (2)	C4A—N1A—C2A—C1A	−120.9 (2)
C4B—N1B—C3B—C2B	110.3 (2)	C4A—N1A—C2A—C3A	112.7 (2)
C4B—N1B—C10B—C11B	−19.1 (4)	C4A—N1A—C10A—C11A	−17.5 (4)
C4B—C5B—C6B—C7B	−0.4 (4)	C4A—C5A—C6A—C7A	0.1 (4)
C5B—C4B—C9B—C8B	−1.8 (4)	C5A—C4A—C9A—C8A	1.6 (4)
C5B—C6B—C7B—C8B	−1.0 (4)	C5A—C6A—C7A—C8A	1.3 (4)
C6B—C7B—C8B—C9B	1.0 (4)	C6A—C7A—C8A—C9A	−1.3 (4)
C7B—C8B—C9B—C4B	0.5 (4)	C7A—C8A—C9A—C4A	−0.1 (4)
C9B—C4B—C5B—C6B	1.8 (4)	C9A—C4A—C5A—C6A	−1.6 (4)
C10B—N1B—C3B—C1B	57.5 (3)	C10A—N1A—C2A—C1A	56.4 (3)
C10B—N1B—C3B—C2B	−68.4 (3)	C10A—N1A—C2A—C3A	−69.9 (3)
C10B—N1B—C4B—C5B	133.1 (2)	C10A—N1A—C4A—C5A	−44.5 (3)
C10B—N1B—C4B—C9B	−48.8 (3)	C10A—N1A—C4A—C9A	137.6 (2)
C10B—C11B—C12B—C13B	−50.5 (3)	C10A—C11A—C12A—C13A	132.2 (3)
C10B—C11B—C12B—C17B	131.9 (3)	C10A—C11A—C12A—C17A	−49.3 (3)
C10B—C11B—B1B—O1B	−0.5 (4)	C10A—C11A—B1A—O1A	1.7 (4)
C10B—C11B—B1B—O2B	176.9 (2)	C10A—C11A—B1A—O2A	−179.8 (2)
C11B—C12B—C13B—C14B	−179.0 (2)	C11A—C12A—C13A—C14A	−179.5 (2)
C11B—C12B—C17B—C16B	179.3 (2)	C11A—C12A—C17A—C16A	179.9 (2)
C12B—C11B—B1B—O1B	−175.6 (2)	C12A—C11A—B1A—O1A	−177.2 (2)
C12B—C11B—B1B—O2B	1.7 (4)	C12A—C11A—B1A—O2A	1.4 (4)
C12B—C13B—C14B—C15B	0.2 (4)	C12A—C13A—C14A—C15A	−1.0 (4)
C13B—C12B—C17B—C16B	1.6 (3)	C13A—C12A—C17A—C16A	−1.6 (3)
C13B—C14B—C15B—C16B	0.8 (4)	C13A—C14A—C15A—C16A	−0.6 (4)
C14B—C15B—C16B—C17B	−0.5 (4)	C14A—C15A—C16A—C17A	1.1 (4)
C15B—C16B—C17B—C12B	−0.6 (4)	C15A—C16A—C17A—C12A	0.1 (4)
C17B—C12B—C13B—C14B	−1.3 (3)	C17A—C12A—C13A—C14A	2.0 (3)
C18B—O1B—B1B—O2B	1.1 (3)	C18A—O1A—B1A—O2A	−1.2 (3)
C18B—O1B—B1B—C11B	178.7 (2)	C18A—O1A—B1A—C11A	177.5 (2)
C18B—C19B—C20B—C21B	−0.9 (4)	C18A—C19A—C20A—C21A	0.1 (4)
C19B—C18B—C23B—O2B	−177.8 (2)	C19A—C18A—C23A—O2A	179.4 (2)
C19B—C18B—C23B—C22B	0.1 (4)	C19A—C18A—C23A—C22A	−1.2 (4)
C19B—C20B—C21B—C22B	0.3 (4)	C19A—C20A—C21A—C22A	−0.2 (4)
C20B—C21B—C22B—C23B	0.5 (4)	C20A—C21A—C22A—C23A	−0.3 (4)
C21B—C22B—C23B—O2B	176.8 (2)	C21A—C22A—C23A—O2A	−179.7 (2)
C21B—C22B—C23B—C18B	−0.7 (4)	C21A—C22A—C23A—C18A	1.0 (4)
C23B—O2B—B1B—O1B	−1.1 (3)	C23A—O2A—B1A—O1A	1.0 (3)
C23B—O2B—B1B—C11B	−178.7 (2)	C23A—O2A—B1A—C11A	−177.8 (2)
C23B—C18B—C19B—C20B	0.7 (4)	C23A—C18A—C19A—C20A	0.6 (4)
B1B—O1B—C18B—C19B	177.0 (2)	B1A—O1A—C18A—C19A	−178.8 (3)
B1B—O1B—C18B—C23B	−0.6 (3)	B1A—O1A—C18A—C23A	1.0 (3)
B1B—O2B—C23B—C18B	0.7 (3)	B1A—O2A—C23A—C18A	−0.3 (3)
B1B—O2B—C23B—C22B	−177.1 (2)	B1A—O2A—C23A—C22A	−179.7 (3)
B1B—C11B—C12B—C13B	124.1 (3)	B1A—C11A—C12A—C13A	−49.1 (3)
B1B—C11B—C12B—C17B	−53.5 (3)	B1A—C11A—C12A—C17A	129.4 (2)
